# Rapid DNA/eDNA‐Based ID Tools for Improved Chondrichthyan Monitoring and Management

**DOI:** 10.1111/1755-0998.70044

**Published:** 2025-09-19

**Authors:** Marcela Maki Alvarenga, Ingrid Vasconcellos Bunholi, Aisni Mayumi C. L. Adachi, Marcelo Merten Cruz, Leonardo Manir Feitosa, Eduarda Valério de Jesus, Maria Eduarda Leda Lopes, Cintia Povill, Daniela Souza, Yan Torres, Antonio Mateo Solé‐Cava, Rodrigo Rodrigues Domingues, Patricia Charvet, Vanessa Paes da Cruz

**Affiliations:** ^1^ CIBIO, Centro de Investigação em Biodiversidade e Recursos Genéticos InBIO Laboratório Associado, Campus de Vairão, Universidade do Porto Vairão Portugal; ^2^ Departamento de Biologia, Faculdade de Ciências Universidade do Porto Porto Portugal; ^3^ BIOPOLIS Program in Genomics, Biodiversity and Land Planning, CIBIO, Campus de Vairão Vairão Portugal; ^4^ CENIMP, Centro Nacional para a Identificação Molecular do Pescado Instituto de Biologia, Universidade Federal do Rio de Janeiro (UFRJ) Rio de Janeiro Brazil; ^5^ Department of Marine Science The University of Texas at Austin, Marine Science Institute Port Aransas Texas USA; ^6^ Departamento de Biologia Estrutural e Funcional Instituto de Biociências, Universidade Estadual Paulista (UNESP), Campus de Botucatu Botucatu Brazil; ^7^ Grupo de Investigação Biológica Integrada, Centro de Estudos Avançados da Biodiversidade Universidade Federal do Pará (UFPA) Belém Brazil; ^8^ Bren School of Environmental Science and Management University of California Santa Barbara California USA; ^9^ Departamento de Oceanografia Biológica Instituto Oceanográfico, Universidade de São Paulo (USP) São Paulo Brazil; ^10^ Instituto Tecnológico Vale—Desenvolvimento Sustentável Belém Brazil; ^11^ Zoological Institute, Department of Environmental Sciences University of Basel Basel Switzerland; ^12^ Programa de Pós‐graduação em Sistemática, Uso e Conservação da Biodiversidade (PPGSis) Universidade Federal do Ceará (UFC) Fortaleza Brazil; ^13^ Departamento de Ciências Biológicas e Ambientais Instituto de Biociências, Universidade Estadual Paulista (UNESP), Campus do Litoral Paulista São Vicente Brazil

**Keywords:** conservation genetics, ddPCR, elasmobranch, environmental DNA, fisheries management, LAMP, Multiplex PCR, qPCR, sharks and rays, species identification

## Abstract

Rapid DNA/eDNA‐based ID tools, which detect specific genetic patterns without requiring sequencing, are essential for biodiversity and wildlife trade monitoring, particularly for species of conservation concern. However, the practical application of these methods remains limited by the availability of standardised protocols, accessibility of resources, and coverage across diverse taxa. This challenge is especially pronounced for Chondrichthyes, a group heavily overexploited due to fishing and illegal trade, and with data scarcity for conservation assessments. Despite their ecological and economic importance, many species lack reference sequences in databases, as well as other molecular data and tools, hindering the development of molecular tools for species identification and trade regulation. This review synthesises the current state of rapid DNA/eDNA‐based ID tools for the detection of chondrichthyan species, including established and emerging methods. It also compiles available taxon‐specific primers to facilitate efficient species identification and recommends the most suitable methods. We identify key gaps in taxonomic and geographic coverage, emphasising the need for further research to expand these tools to under‐represented species and regions. Additionally, we highlight the importance of integrating genetic approaches into enforcement frameworks to enhance conservation strategies and regulatory compliance. By providing an accessible reference for time‐ and cost‐effective genetic monitoring, this work will support evidence‐based decision‐making and improve the practical application of rapid DNA/eDNA‐based ID tools in the conservation and management of Chondrichthyes species worldwide.

## 1 Introduction

Rapid DNA/eDNA‐based ID tools are genetic methods used for taxon identification that detect specific DNA patterns without the need for nucleotide sequencing. These techniques allow fast and efficient identification when morphological identification is impractical and can thus play a crucial role in biodiversity conservation by contributing to monitoring and enforcement (Shivji et al. 2002; Jiménez‐Pérez et al. 2025; Ramanan et al. 2025). These methods are particularly valuable in resource‐limited geographic regions, as applied to healthcare services (Carvajal Aristizabal et al. 2024; Gulley and Morgan 2014). Rapid species identification helps to improve the enforcement of fisheries and trade regulations and may be especially valuable for species at high extinction risk (De‐Franco et al. 2012; Alvarenga, Bunholi, et al. 2024; Chapman et al. 2025), such as the Chondrichthyes (sharks, rays and chimaeras). These include species that are among the most threatened vertebrates in the world, primarily due to the high value of their fins in Asian markets, coupled with a high incidence of non‐targeted catches (i.e., bycatch) and increasing global demand for shark and ray meat, all leading to overfishing (Dulvy et al. 2024; Niedermüller et al. 2021; Oliver et al. 2015; Pacoureau et al. 2023). As a result, chondrichthyan fisheries have expanded from pelagic to coastal and deep‐sea fisheries (Worm et al. 2024), accounting for a decline of over 50% in Chondrichthyes biodiversity (Dulvy et al. 2024).

Due to local, regional and international demand for their products and byproducts (meat, fins, liver oil, cartilage, etc.), shark overfishing has become a significant concern, prompting the implementation of legislation at multiple levels to regulate shark fisheries and trade (Dent and Clarke 2015; Dulvy et al. 2024; Niedermüller et al. 2021). Many shark and ray species have broad geographic ranges and are exploited by multiple nations, which highlights the need for transboundary cooperation (Dulvy et al. 2024). However, regulations governing chondrichthyan fisheries vary significantly across countries, reflecting diverse management and conservation approaches (Worm et al. 2024). Some nations enforce total fishing bans, set quotas, or establish no‐take areas and marine sanctuaries, while others focus on protecting specific threatened species (Akhilesh et al. 2023; Alvarenga, Bunholi, et al. 2024; Jaiteh et al. 2016; Ward‐Paige and Worm 2017). Nonetheless, the effectiveness of these measures is often compromised by enforcement challenges, including limited financial and human resources, which undermine national and international compliance and weaken fisheries management (Davidson et al. 2022; Fischer et al. 2012; Hammerschlag and Sims 2024).

The number of Chondrichthyes listed under the Convention on International Trade in Endangered Species of Wild Fauna and Flora (CITES) has now surpassed 170 species, with most of them listed under Appendix 1 (CITES 2025), requiring certificates of sustainable harvest for international trade. Despite these frameworks, many countries still struggle to fully implement or comply with international agreements, resulting in inconsistent regulations and continued threats to chondrichthyan populations (Dulvy et al. 2021; Hammerschlag and Sims 2024). It is also important to emphasise that illegal fishing activities and harmful fisheries subsidies in several countries hinder the development of effective management strategies and limit access to reliable data for fisheries assessments (Dulvy et al. 2024; Garcia 2024). The widespread distribution and migratory nature of some species pose additional challenges for monitoring and management efforts (Das et al. 2022; Osuka et al. 2025). Furthermore, morphological similarities among species and common fishing practices—such as the onboard removal of head, fins and viscera with only processed body parts landed (Alvarenga et al. 2021; Domingues et al. 2013; Shivji et al. 2005)—make species identification difficult when based on morphological characters.

In this context, genetic‐based methods have become increasingly valuable for identifying Chondrichthyes species for trade monitoring and improving species management and conservation (Cardeñosa et al. 2024; Dudgeon et al. 2012; Ramanan et al. 2025; Kasana et al. 2025). These approaches range from rapid techniques targeting specific taxa—such as PCR–RFLP (PCR–restriction fragment length polymorphism), multiplex PCR, qPCR (quantitative PCR), ddPCR (droplet digital PCR) and LAMP (loop‐mediated isothermal amplification)—to more advanced sequencing methods that provide broader community‐level information, including DNA barcoding and metabarcoding (Abercrombie et al. 2005; Bunholi et al. 2018; Dudgeon et al. 2012; Mariani et al. 2021; Prasetyo et al. 2023; Maiello et al. 2025; Dunn et al. 2025). For a detailed overview of sequencing‐based identification tools vs. rapid identification tools, see Box 1.

BOX 1 **Sequencing‐based ID methods vs. rapid DNA/eDNA‐based ID tools.**

Sequencing‐based approaches

DNA barcoding amplifies and sequences a short, standardised gene region, offering a straightforward and potentially highly accurate method for identifying species (Abdi et al. 2024; Antil et al. 2023; Hebert et al. 2003). However, this approach is limited in handling complex, multi‐species samples – particularly when using universal primers on unknown samples instead of taxon‐specific ones for presence/absence detection – and relies heavily on comprehensive reference databases, which remain incomplete for many hyperdiverse or understudied taxa (Alvarenga, Bunholi, et al. 2024; Antil et al. 2023). In addition, barcoding can become expensive and time‐consuming when used in large‐scale biodiversity assessments or routine monitoring of illegal trade, making it harder to keep up with high‐throughput demands (Antil et al. 2023; Muhammad Tahir and Akhtar 2016). Moreover, this method requires access to sequencing facilities in addition to DNA extraction and PCR infrastructure, which can pose logistical and financial challenges in low‐resource settings (Muhammad Tahir and Akhtar 2016).

To overcome some of these challenges, DNA metabarcoding extends the barcoding framework by utilising high‐throughput sequencing (HTS) to simultaneously detect multiple species within complex or environmental samples, making it particularly valuable for ecological research, environmental monitoring and food content screening (Cristescu 2014). While metabarcoding offers significant advantages over traditional barcoding, it is important to acknowledge some methodological challenges documented in the literature, including potential amplification biases associated with the use of universal primers (Leray and Knowlton 2017; Keck et al. 2023; van der Loos and Nijland 2021). Some of these limitations can be tackled using shotgun metagenomics, a high‐throughput technique that skips the PCR step and sequences all the DNA present in a sample. This can help reduce some of the biases seen in PCR‐based methods, improve taxonomic resolution and broaden the range of species that can be detected (Manu and Umapathy 2023; Shokralla et al. 2012). Moreover, tools to recover and assemble mitochondrial genomes from HTS data are getting better and easier to use [e.g., MitoFinder (Allio et al. 2020)], which enables the reconstruction of complete mitogenomes directly from sequencing reads. This is particularly advantageous when working with degraded DNA or when suitable primers are unavailable. Compared to standard DNA metabarcoding markers such as COI, full mitochondrial genomes provide access to additional loci with higher taxonomic resolution for Chondrichthyes (e.g., ND2), enhancing the detection of closely related or cryptic species (Alvarenga, D'Elia, et al. 2024). Mapping reads to the mitochondrial genome also provides more accurate quantification of species' DNA (Ji et al. 2020). Nevertheless, shotgun sequencing is usually more expensive and generally requires better‐quality DNA compared to PCR‐based approaches, which are more tolerant of degraded or low‐concentration samples (Lamoureux et al. 2022; Latz et al. 2022). It is possible to achieve with degraded or low‐concentration samples (e.g., short‐read sequencing in ancient DNA studies), but doing so often means following more specialised protocols and more sequencing effort, which raises costs and complexity (Colella et al. 2020). Finally, both shotgun sequencing and metabarcoding call for specialised bioinformatics skills and considerable computational power, which are unavailable in many settings (Lamoureux et al. 2022).

Rapid DNA/eDNA‐based ID tools

In contrast, rapid DNA/eDNA‐based ID tools (e.g., taxon‐specific PCR, multiplex PCR, taxon‐specific qPCR and ddPCR) are relatively fast and low‐cost alternatives that can be used with more widely accessible lab infrastructure. If well‐established, these methods often do not require sequencing and can detect specific DNA markers rapidly. Commonly used rapid methods are great options for fieldwork, enforcement operations, or quick screenings in remote or low‐resource areas due to their simplicity, cost‐effectiveness and portability (Böhme et al. 2019; Cardeñosa et al. 2018; Langlois et al. 2021). Nonetheless, rapid DNA/eDNA‐based ID tools require prior knowledge of the target DNA and may rely on very specific primers or probes (e.g., multiplex PCR, ddPCR, taxon‐specific qPCR), meaning that they are most useful for well‐characterised species (Böhme et al. 2019). In this context, “well‐characterised species” refers to taxa for which multiple, high‐quality reference sequences are available in public databases such as GenBank and BOLD, encompassing both intraspecific variation and closely related or sympatric taxa. The inclusion of phylogenetically proximate species in the reference dataset enhances assay specificity by enabling more robust *in silico* validation, thereby reducing the risk of non‐target amplification and subsequent misidentification (Langlois et al. 2021; Böhme et al. 2019; Kim et al. 2023). Methods such as rep‐PCR and RT‐HRM‐PCR are excluded from this discussion due to their low repeatability and limited suitability for standardised molecular identification tasks. Finally, rapid DNA/eDNA‐based ID tools can also be sensitive to contamination or inhibitors like humic acids (common in eDNA samples) or ethanol (from preserved tissues), which may lead to false positives or negatives (Mauvisseau et al. 2019; Rieder et al. 2024).

Applications and standardization of rapid DNA/eDNA‐based ID assays

Despite their limitations, rapid DNA/eDNA‐based ID tools can be highly effective when applied in appropriate contexts. These tools can deliver results within approximately one to a few hours, making them valuable in situations where timely decision‐making is essential and sample processing speed can make a significant difference (Böhme et al. 2019; But et al. 2020; Cardeñosa et al. 2018), such as during customs wildlife shipment checking, early detection of invasive species, or monitoring rare and threatened species. These quick tests can act as a first step, and their results can later be confirmed through more detailed methods like DNA barcoding, adding a layer of reliability. Furthermore, the sensitivity of ddPCR and taxon‐specific qPCR is often higher than standard metabarcoding, making it an excellent option for detecting rare or low‐abundance species (Doi et al. 2015). This is because targeted assays (e.g., taxon‐specific qPCR/ddPCR) are less affected by amplification bias, which occurs in metabarcoding when high‐abundance DNA outcompetes low‐concentration templates during PCR (Guri et al. 2024; Collins et al. 2019; Wood et al. 2019). Such bias may lead to non‐detection of rare species, especially when universal primers preferentially amplify common taxa. Therefore, *in silico* evaluation of primer specificity and sensitivity using tools such as Primer‐BLAST (Ye et al. 2012) or ecoPCR (Ficetola et al. 2010) is essential for the reliable use of rapid DNA/eDNA‐based identification tools in Chondrichthyes. Practitioners should evaluate the availability and completeness of reference databases for target taxa, and ensure that primers do not amplify non‐target species. It is also important to assess potential gaps in the databases that could affect taxonomic resolution. Positive and negative controls, when feasible, confirmatory sequencing, performed on a subset of samples, can be used to initially validate primer efficiency and specificity, helping to minimise false positives (Bustin 2004; Klymus et al. 2020; Langlois et al. 2021).

Overall, the best method depends on the context and goals of the study. A tiered or integrated strategy, starting with rapid diagnostics (e.g., LAMP, taxon‐specific qPCR) and following up with sequencing (e.g., Sanger or batch amplicon sequencing to save costs), can provide a cost‐effective and scalable strategy for molecular monitoring and enforcement. New portable sequencing tools, like nanopore platforms, are becoming more promising and may soon help bridge the gap between field‐friendly and high‐resolution sequencing methods (Johri et al. 2019; Truelove et al. 2019; Srivathsan et al. 2018). Ultimately, method selection must balance resolution, cost and logistical feasibility, and should always be adapted to the target taxa and application context.

Recent advancements in molecular techniques, such as extracting DNA from environmental samples (environmental DNA or eDNA) combined with taxon‐specific PCR or metabarcoding, now allow the detection of Chondrichthyes species directly from water samples (Boussarie et al. 2018; Mariani et al. 2021). This approach offers a powerful alternative to traditional, costly and labour‐intensive survey methods like underwater visual censuses (UVCs) and baited remote underwater video systems (BRUVS), eliminating the need for specialised equipment and extensive fieldwork. Boussarie et al. (2018) detected 44% more shark species from eDNA metabarcoding compared to UVCs and BRUVS, including the identification of previously unobserved shark species in the New Caledonian Archipelago. Environmental DNA can also be integrated into routine fisheries activities by collecting water dripping from fishing nets (Albonetti et al. 2023; Boussarie et al. 2018; Lafferty et al. 2018). A novel device, the metaprobe, has been developed to collect eDNA directly within active fishing gear (Maiello, Bellodi, et al. 2024). This technique has proven effective not only for monitoring fish catches, including elasmobranchs, but also for large‐scale assessments of fish assemblages (Maiello, Bellodi, et al. 2024; Maiello, Talarico, et al. 2024). Beyond species detection, eDNA offers potential for tracking distributional changes and seasonal patterns (Budd et al. 2023). The most informative assays employ metabarcoding, although eDNA metabarcoding methods remain expensive, especially for Global South countries (Bunholi et al. 2023; Hirsch et al. 2024). Cost constraints are particularly significant in large‐scale monitoring programmes requiring high sample throughput, though efforts are underway to develop more affordable yet accurate alternatives (Johri et al. 2019; Theissinger et al. 2023).

While various DNA‐ and eDNA‐based methods have been developed to identify sharks and rays, selecting the most appropriate approach requires consideration of the study's purpose (e.g., species‐specific detection vs. community‐level assessment), objective (e.g., law enforcement, monitoring, statistical analysis, or scientific research) and regional context (e.g., funds allocation per region). Furthermore, the availability of pre‐existing tools can also improve the time‐ and cost‐effectiveness of trade inspections, conservation policies, or scientific projects. Among these methods, rapid DNA/eDNA‐based ID tools stand out for their speed, cost‐effectiveness (in both DNA and eDNA applications; Cardeñosa et al. 2018; Langlois et al. 2021) and sensitivity (particularly in eDNA contexts; Bylemans et al. 2019; McColl‐Gausden et al. 2023) compared to barcoding and metabarcoding. Nevertheless, their potential remains underutilised in trade monitoring compared to sequencing‐based technologies (Cardeñosa et al. 2018). Therefore, rapid DNA/eDNA‐based ID tools (see Box 2 for a detailed overview) for genetic‐based monitoring of Chondrichthyes were the central focus of this review.

BOX 2 **Practioners' guide to rapid DNA/eDNA‐based ID tools.**

Several rapid DNA and eDNA tools have been successfully applied in Chondrichthyes studies, contributing significantly to conservation and monitoring efforts. However, no single method is universally optimal for species identification. The best choice depends on the research goal, sample type and logistical context. For instance, when working with tissue DNA, researchers or enforcement agents aiming to identify specific known species (e.g., guitarfishes, hammerheads, or pelagic sharks) from regional fisheries may opt for well‐established rapid ID methods such as multiplex PCR, taxon‐specific PCR, or PCR–RFLP, which are already validated for these groups in certain regions [e.g., endemic guitarfish from the South Atlantic (De‐Franco et al. 2010), population of pelagic sharks from the Eastern Tropical Pacific (Caballero et al. 2012)] (see Table S1 for available sets and target taxa). In contrast, if the range of potential species is broader or unknown, sequencing‐based approaches such as DNA barcoding (using markers like the COI mini‐barcode for degraded samples [Cardeñosa et al. 2017; Wannell et al. 2020], NADH2 [Leurs et al. 2023; Vella et al. 2017], or 12S rRNA [e.g., Miya et al. 2015]) may offer better resolution. These allow species assignment without prior assumptions and are particularly useful for surveillance of illegal trade or biodiversity assessments.

The same logic applies to eDNA‐based approaches. If the goal is to detect or monitor a specific species or a small group of targets, practitioners have the option to use existing primer and probe sets (see Tables S3 and S4 for an overview) or design new sets tailored to their needs. They can choose the most appropriate technique based on the complexity of the samples and the requirements for detection, such as taxon‐specific qPCR for identifying specific taxa with higher specificity, or ddPCR, which is particularly useful for detecting rare taxa. However, for community‐level monitoring, tools like DNA metabarcoding are more appropriate due to their capacity to detect multiple species simultaneously.

To assist in method selection, Table 1 provides a comprehensive overview of rapid DNA/eDNA‐based ID tools tested for Chondrichthyes, along with their sample compatibility, strengths, limitations and current applicability for Chondrichthyes. The second half of this box outlines future directions, highlighting portable and field‐deployable tools that may become increasingly relevant. Together, this guidance can help practitioners choose the most appropriate method for their needs while contributing to the continued advancement of rapid molecular tools for shark and ray conservation.

**TABLE 1:** Overview of rapid DNA/eDNA‐based ID tools for Chondrichthyes species identification, offering practitioners guidance on selecting the most suitable approach for their specific needs.

Technique name	Summarised explanation	Practical applications	Pros, cons and recommendations	Use for Chondrichthyes ID
Taxon‐Specific PCR	Amplifies DNA from a specific taxon using a primer pair tailored for that taxon, with results visualised through gel or capillary systems. **Interpretation**: Band at expected size = detection; no band = absence or failed reaction. **Controls**: Positive and negative controls are recommended to validate the accuracy and reliability of the reaction.	Assigning unidentified tissue parts to a specific taxon or testing for the presence of a specific taxon in environmental samples and complex food mixtures.	**Pros:** Highly targeted; cost‐effective; non‐target DNA unlikely present in tissue samples; suitable for well‐characterised taxa, and with reference databases of sympatric and closely related taxa also available. **Cons:** Risk of non‐target amplification with closely related taxa; low specificity and resolution for eDNA applications; often requires sequencing a subset of targets for validation. **Recommendations:** During the development of the assay, ensure careful primer optimisation and rely on comprehensive reference databases to reduce the likelihood of non‐target amplifications. For higher specificity, consider taxon‐specific qPCR.	**Tissue DNA:** Clarke et al. (2006); Doukakis et al. (2011); Sodré et al. (2024) **eDNA:** Bonfil et al. (2021); Bonfil et al. (2024); Ferretti et al. (2024); Jenrette et al. (2023); Lim and Then (2022)
Multiplex PCR	A variation of PCR that allows the addition of multiple taxon‐specific primers in a single reaction. Each pair of primers will amplify a distinct DNA fragment size, enabling the testing of multiple taxa in one assay. The amplified products can be visualised through gel or capillary electrophoresis, returning one taxon per reaction. **Interpretation**: Band at expected size = taxon detected; no band = absence or failed reaction. **Controls**: Positive and negative controls are recommended.	Allows rapid taxa detection, ideal for routine trade inspections and monitoring programmes.	**Pros:** Screens for multiple target taxa in one reaction; efficient for known targets; cost‐effective for routine screening; suitable for identifying samples from fisheries with few target taxa (e.g., guitarfishes). **Cons:** Non‐specific amplification may occur; development can be time‐consuming. **Recommendations:** When available, use validated assays or primers from established databases; avoid redundant assay development by checking existing literature and databases first, but be cautious when using a protocol developed in a different region due to intraspecific polymorphism; for higher specificity and on‐site, prefer multiplex qPCR; for closely related taxa, opt for PCR–RFLP.	**Tissue DNA**: Abercrombie et al. (2005); Aguilar‐Rendón et al. (2020); Caballero et al. (2012); Chapman et al. (2003); De‐Franco et al. (2012); De‐Franco et al. (2010); Domingues et al. (2013); Farrell et al. (2009); Ferrette et al. (2019); Hernández et al. (2010); Hoelzel (2001); Magnussen et al. (2007); Manzanillas Castro and Acosta‐López (2022); Marino et al. (2014); Mendonça et al. (2010); Nachtigall et al. (2017); Pank et al. (2001); Pinhal et al. (2012); Shivji et al. (2002); Shivji et al. (2005)
PCR–RFLP	Amplified DNA is digested by restriction enzymes, generating taxon‐specific fragment patterns. Fragments are separated and visualised by gel or capillary electrophoresis. **Interpretation**: Taxon identification is achieved by comparing the observed restriction fragment pattern to established reference profiles, based on the specific positions of restriction enzyme recognition sites, which determine the size and number of resulting fragments per taxon. **Controls**: Positive and negative controls recommended	Fast taxa differentiation, especially for closely related taxa or those with limited genetic divergence, as restriction sites require fewer diagnostic SNPs than primers to achieve specificity in conserved regions.	**Pros:** Cost‐effective; simple implementation in basic labs; cheaper for multi‐target testing when one enzyme can distinguish multiple taxa; easier and cheaper to develop than multiplex PCR. **Cons:** Limited by the availability of informative taxon‐specific restriction sites; lower sensitivity than probe/primer‐based methods, as taxa ID depends on digestion patterns rather than precise primer/probe binding; slower due to two separate steps (PCR and digestion) and two visualisation steps. **Recommendations:** Assay development should test multiple enzymes (in silico to shortlist the most promising candidates and in vitro to confirm the one yielding the most diagnostic banding patterns).	**Tissue DNA**: Canfield and Bowen (2021); Cannas et al. (2010); Chan et al. (2003); Ferrito et al. (2019); Mariguela et al. (2009); Mendonca et al. (2009); Schmidt et al. (2015)
Taxon‐Specific qPCR	Monitors DNA amplification in real‐time during each cycle using fluorescent dyes or target‐specific probes. Fluorescence increases as DNA amplifies, being visualised through the Ct (cycle threshold). **Interpretation**: Lower Ct = more starting DNA; higher Ct = less starting DNA; no Ct = absence of DNA or inhibition. **Controls**: Positive, negative and inhibition controls are recommended.	Effective for taxa identification and quantification in mixed samples (e.g., food) and eDNA biodiversity monitoring. Portable machines enable on‐site identification.	**Pros**: High sensitivity and specificity with taxon‐specific probes; provides quantitative data instead of only qualitative PCR data; faster than traditional PCR; portable devices enable field application. **Cons**: Less sensitive for rare taxa compared to ddPCR for eDNA applications; prone to primer‐dimers and secondary structures; requires more expensive equipment than taxon‐specific PCR. **Recommendations:** For tissue samples, consider affordable options like multiplex PCR or PCR–RFLP; for rare taxa in eDNA, opt for ddPCR.	**Tissue DNA**: Hwang et al. (2015); Kim et al. (2018). **eDNA**: Budd et al. (2021); Budd et al. (2023); Cooper et al. (2021); Cooper et al. (2022); Dunn et al. (2023); Faure et al. (2023); Gargan et al. (2017); Postaire et al. (2020); Weltz et al. (2017)
Multiplex qPCR	Combines quantitative PCR with multiplexing by using multiple primer sets, each with a distinct amplicon size. An intercalating binding dye can be added (e.g., SYBR Green, usually in tissue DNA) or specific probes often labelled with distinct fluorophores (usually in eDNA), enabling the simultaneous testing of multiple targets within a single reaction. **Interpretation**: Ct interpretations are as for qPCR, with the presence of distinct fluorescence curves of different sizes if testing complex samples. **Controls**: Positive, negative and inhibition controls are recommended.	Suitable for multi‐taxa detection and quantification, from tissue and complex samples.	**Pros:** Similar advantages of qPCR, but also enabling testing the presence of multiple targets in one reaction; faster than Multiplex PCR and with the possibility of application on‐site. **Cons:** Complex and time/cost consuming assay design; risk of non‐specific amplification when not adding specific probes also; more expensive than Multiplex PCR. **Recommendations:** Use reliable primer and probe databases and optimise reactions; for tissue DNA/non‐complex samples that do not require on‐site qPCR machines, consider cheaper alternatives (e.g., Multiplex PCR, PCR–RFLP).	**Tissue DNA**: Cardeñosa et al. (2018); Cardeñosa et al. (2023). **eDNA**: van Rooyen et al. (2021)
Droplet Digital PCR (ddPCR)	A highly sensitive method for detecting and quantifying DNA by partitioning samples into thousands of droplets for amplification, each undergoing independent amplification. Target detection is based on probe‐specific fluorescence signals. **Interpretation**: Fluorescent‐positive droplets = target DNA detected. No fluorescence = absence or failure. More fluorescent‐positive droplets = more DNA. **Controls**: Positive, negative and inhibition controls are recommended.	Ideal for quantitative detection of rare taxa or low‐abundance targets in complex samples.	**Pros:** Extremely high sensitivity for rare targets; resistant to inhibitors; no need for standard curves or replicates; simpler to operate than qPCR. **Cons:** High cost; still requires specialised equipment and trained personnel. **Recommendations:** For abundant species, prefer qPCR cheaper alternatives.	**eDNA**: Drymon et al. (2021); Lafferty et al. (2018); Lehman et al. (2020); Lehman et al. (2022); Schweiss et al. (2020)
Loop‐mediated Isothermal Amplification (LAMP)	Amplifies DNA at a constant temperature using a set of taxon‐specific primers. from multiple loci Amplification induces changes in the reaction mixture's chemistry, resulting in visible colour change or turbidity when using pH‐ or metal ion‐sensitive dyes. Detection can be performed directly in the reaction tube or on paper‐based devices. **Interpretation**: Visible change = detection; no change = absence. **Controls**: Positive/negative controls required.	Ideal for rapid taxa identification, particularly suited for fieldwork or on‐site identification.	**Pros:** Very fast (< 1 h); operates at constant temperature; suitable for field and low‐resource settings; detection can be visual (e.g., colour change), interpretable by eye without equipment. **Cons:** Possible to multiplex, but with limited capacity; complex primer design; higher risk of false positives due to potential primer non‐specific amplification and reliance on sensitive chemical reactions. **Recommendations:** Rely on comprehensive reference databases to reduce the likelihood of non‐target amplifications; validate carefully to reduce false positives.	**Tissue DNA**: But et al. (2020); Lin et al. (2021); Tiktak et al. (2024), Jiménez‐Pérez et al. (2025)
Repetitive sequence‐based PCR (rep‐PCR)	Utilises primers that bind to repetitive DNA elements dispersed throughout the genome to generate taxa‐specific amplification patterns. Resulting fragments are separated and visualised by gel or capillary electrophoresis. **Interpretation**: Taxa identification is based on matching the band pattern to a validated reference profile. **Controls**: Positive and negative controls are recommended; a reference database of patterns is essential.	Used for distinguishing closely related taxa.	**Pros:** Low cost; simple development; suitable for taxa with different amounts of repetitive DNA; fewer primers and no enzymes required. **Cons:** Sensitive to DNA quality and quantity; limited by repetitive sequence availability; low reproducibility; does not factor in intraspecific variation; no longer used. **Recommendations:** Consider more established rapid methods when appropriate; for closely related taxa, prefer PCR–RFLP.	**Tissue DNA**: Pinhal et al. (2008); Pinhal et al. (2009)
Real‐Time High‐Resolution Melting PCR (RT‐HRM‐PCR)	Combines qPCR with high‐resolution melting analysis to discriminate DNA sequences based on differences in melting curve profiles. **Interpretation**: Unique melting curve profiles are used to differentiate taxa. Closely related taxa may require sequencing for confirmation. **Controls**: Positive and negative controls are required; reference melt profiles and high‐quality DNA are essential.	Not widely applied.	**Pros:** Do not require probes. **Cons:** Requires precise temperature control and high‐quality DNA; not reliable with degraded or complex samples; limited validation and usage. **Recommendations:** Not recommended; prefer more modern and accurate techniques for most applications.	**Tissue DNA**: Morgan et al. (2011)
FASTFISH‐ID	Closed‐tube, PCR‐based DNA identification method that uses a universal set of primers and fluorescently labelled probes. The probes hybridise to the amplified target barcode region (e.g., COI), generating a species‐specific fluorescent signature recorded in real time. **Interpretation**: The resulting fluorescence curves form a unique pattern for each species, which is compared against a reference database; machine learning algorithms can assist in discriminating closely related taxa. **Controls**: Positive, negative and reference pattern profiles.	One‐step reaction DNA‐based taxa species identification for fisheries monitoring and enforcement.	**Pros:** Easy and rapid results; works with degraded samples; can identify species in degraded samples; enables on‐site identification; fully developed and commercially available with an extensive fish reference database. Minimal technical expertise required. Uses the same universal primer/probe set for all species; only the reference database needs to be expanded for new taxa. **Cons**: Limited to species in the reference database; very limited chondrichthyan coverage; poor performance with mixed DNA samples; requires proprietary equipment, reagents and internet access; relatively costly. **Recommendations:** For chondrichthyan enforcement, first expand and validate local reference profiles with the help of confirmatory methods (e.g., DNA barcoding) for species not yet covered.	**Tissue DNA**: Prasetyo et al. (2023)

*Note:* This includes concise explanations, practical applications, main limitations with suggested solutions, and references to studies that have applied each technique, including the target sample types.

- On‐site identification

Although most techniques are laboratory‐based, some can be adapted for on‐site identification. For instance, LAMP facilitates rapid species detection in the field without requiring specialised equipment, as results can be visually interpreted through a colour change (Lin et al. 2021; Tiktak et al. 2024). In contrast, qPCR requires portable devices, such as the GENECHECKER Ultra‐Fast Real‐Time PCR system (Kim et al. 2018). Another promising innovation for on‐site identification is the FASTFISH‐ID, a commercial platform combining COI DNA barcoding with fluorescent probes to convert specific DNA sequences into unique fluorescent signatures (ECOLOGENIX 2025; Naaum and Hanner 2015). This technique has successfully identified 43 bony fish species (Naaum and Hanner 2015) and 23 Chondrichthyes species (Prasetyo et al. 2023). Furthermore, FASTSHARK‐ID is currently under development, aiming to identify approximately 150 Chondrichthyes species using the same probe‐based technology developed in FASTFISH‐ID (ECOLOGENIX 2025). Although not included as rapid DNA/eDNA‐based ID tools in our systematic review, the handheld MinION sequencer is another portable tool that has been used for Chondrichthyes identification in both eDNA (Truelove et al. 2019) and tissue DNA applications (Johri et al. 2019). This nanopore‐based device generates long‐read sequences in real time, illustrating that portable sequencing platforms may increasingly be considered part of the rapid molecular toolkit in future applications.

- Complex samples

In addition to tissue DNA and eDNA applications, there is potential for identifying complex samples, such as processed food products like crab cakes, which may contain ray meat (Alvarenga 2020), and for addressing shark‐related incidents with humans (Martin et al. 2024). Techniques adopted for eDNA analysis are often the most effective for such scenarios. For example, shark‐related incidents with humans have been analysed using DNA from surface swabs of bite marks and processed via qPCR with taxon‐specific primers (Martin et al. 2024). Similarly, multiplex PCR and taxon‐specific PCR could confirm the presence of specific organisms in mixed samples, though no rapid DNA/eDNA‐based ID tools have yet been used to detect Chondrichthyes added to mixed food samples.

We conducted a comprehensive global analysis of rapid DNA/eDNA‐based ID tools for the identification of Chondrichthyes species to identify patterns and trends in their applications. We also aimed to provide an accessible resource for practitioners to facilitate the integration of time‐ and cost‐effective genetics assays into shark, ray and chimaera conservation policies. Specifically, primers and associated rapid DNA/eDNA‐based ID tools available for Chondrichthyes worldwide were searched to (1) compile an accessible list of resources published from January 2000 to March 2025 that can contribute to the rapid genetic identification of Chondrichthyes, (2) analyse research trends by country, taxonomic focus and the evolution of rapid DNA/eDNA‐based ID tools over time and (3) discuss the applicability of rapid DNA/eDNA‐based ID tools for conservation and management.

## 2 Research Overview

A systematic literature review was carried out on Web of Science, using targeted keywords to identify studies that implemented rapid DNA/eDNA‐based ID tools for Chondrichthyes research (Appendix S1, Table S1). A rigorous and comprehensive collection was attained by adhering to the Reporting Standards for Systematic Evidence Syntheses (ROSES; Haddaway et al. 2018) and applying the snowball method, examining reference and citation lists for additional relevant studies (Wohlin 2014).

An easy‐to‐use resource for researchers and practitioners was obtained by extracting key information from the final dataset (Appendix S2, Table S2), including types of rapid DNA/eDNA‐based ID tools applied (e.g., multiplex PCR, ddPCR), target sample (i.e., eDNA or tissue DNA), geographic distribution of studies and taxonomic range (i.e., Chondrichthyes species for which DNA tools are available). The dataset was further split into laboratory‐based tools (Appendix S2, Table S3) and on‐site identification methods (Appendix S2, Table S4). Additionally, a comprehensive list of primers (Appendix S2, Table S5—taxon‐specific and Appendix S2, Table S6—universal) and probes (Appendix S2, Table S7) was compiled. We considered taxon‐specific primers as those targeting distinct groups within Chondrichthyes, such as order (e.g., Carcharhiniformes), family (e.g., Carcharhinidae), genus (e.g., *Carcharhinus*) and species (e.g., *Carcharhinus falciformis* ). Metadata extraction, synthesis and data visualisation were performed in R version 4.2.1 (R Core Team 2021). Full methodological details are provided in Appendix S1, and all compiled metadata and accompanying scripts are available on GitHub (https://github.com/Bunholi/DNA_rapid_tools).

## 3 The Global Status of Rapid DNA/eDNA‐Based ID Tools for Chondrichthyes

**FIGURE 1:**
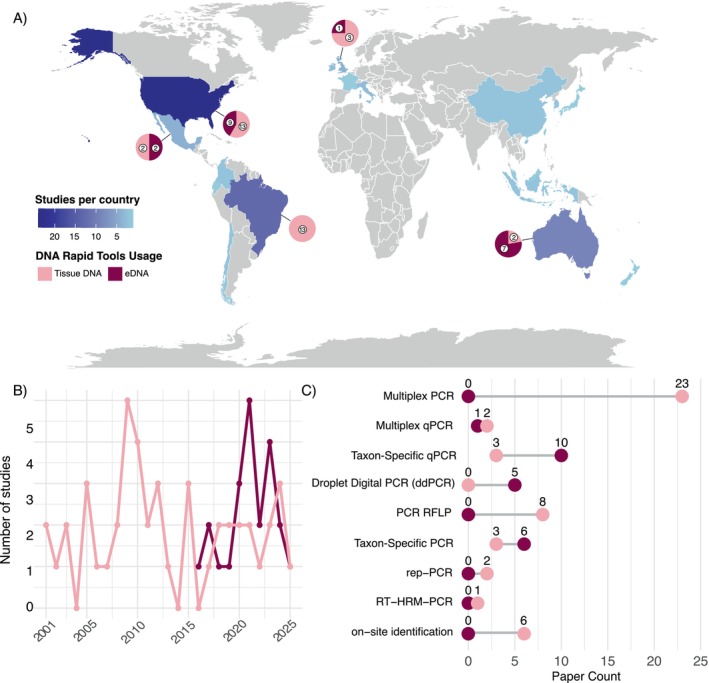
Overview of studies employing rapid DNA/eDNA‐based ID tools for Chondrichthyes identification in tissue and environmental DNA (eDNA) applications. (A) Global distribution of studies, mapped by the locations of first and last authors. Note that author locations do not necessarily reflect the geographic areas where samples were collected. Pie charts indicate the proportional use of tissue DNA and eDNA applications. (B) Temporal distribution of studies for each application. (C) Comparative distribution of studies employing different rapid DNA/eDNA‐based ID tools across both tissue DNA and eDNA applications. Tools categorised as “on‐site identification” include LAMP and FASTFISH‐ID. For more details on each tool, see Box 2.

In total, 68 studies using rapid DNA/eDNA‐based ID tools were compiled from January 2000 to March 2025, comprising 46 studies for tissue DNA tools and 22 studies for eDNA. The geographic distribution of rapid DNA/eDNA‐based ID tools for Chondrichthyes research remains uneven. The United States leads in tool development and application (32.35%), followed by Brazil (19.12%). Nonetheless, Brazil's efforts are currently limited to tissue DNA applications, with tools for eDNA yet to be adopted (Figure 1A). Key countries in the fin trade (e.g., China and Indonesia) and major exporters of shark and ray meat (e.g., Portugal, Spain and Argentina) demonstrate limited development and application of rapid DNA tools for trade monitoring (Niedermüller et al. 2021). It is important to note that the country producing a study does not necessarily source samples from its own territory [e.g., tools developed in the United States have been applied to fin trade samples in Asia (e.g., Cardeñosa et al. 2018; Clarke et al. 2006) (Appendix S2, Table S2)]. Nonetheless, the uneven geographic distribution remains consistent when comparing the location where the rapid DNA/eDNA‐based ID tools were developed to the origin of the samples to which they were applied (Appendix S1, Figure S1).

Expanding the development of rapid DNA/eDNA‐based ID tools to additional regions and associated taxa, particularly in emerging economies, is critical for species conservation. Nevertheless, limited resources and funding in these areas often hinder effective wildlife monitoring and enforcement efforts (Alvarenga, D'Elia, et al. 2024; Booth et al. 2023). Regions with high endemism represent vital hotspots for Chondrichthyes conservation, such as Australia (Last and Stevens 2009), with 12.85% of studies developing rapid DNA/eDNA‐based ID tools and 18.57% utilising samples from Australian waters to apply the tools—primarily for eDNA. Other areas of high endemism, such as Indonesia (Briggs 2003; White and Kyne 2010) and Madagascar (Lucifora et al. 2011), have not advanced much in the development and application of rapid DNA/eDNA‐based ID tools (Figure 1A), underscoring the urgent need for targeted research efforts in biodiversity hotspots.

The earliest documented use of rapid DNA/eDNA‐based ID tools for chondrichthyes monitoring dates to 1998, focusing on tissue DNA applications, particularly for trade regulation (Heist 1998). The use of rapid DNA/eDNA‐based ID tools peaked between 2009 and 2010 before experiencing a decline post‐2013 (Figure 1B). A resurgence in 2023 has been observed, likely driven by advancements in technologies such as portable PCR machines and LAMP‐based tools, which enable rapid and accurate on‐site identifications (Lin et al. 2021; Prasetyo et al. 2023; Tiktak et al. 2024). In contrast, the adoption of rapid eDNA‐based ID tools for presence–absence detection, such as taxon‐specific PCR (also known as taxon‐specific singleplex PCR) and taxon‐specific qPCR (also known as singleplex qPCR), started in 2016 for Chondrichthyes, reached a peak in 2021 with the inclusion of ddPCR, and continues to expand. This trend reflects a broader shift towards non‐invasive methods for biodiversity monitoring, critical for threatened species research (Deiner et al. 2017; Duarte et al. 2023). eDNA tools have enabled remarkable discoveries, such as detecting rare species in the wild, including those previously thought to be extinct in their natural range (Lafferty et al. 2018; Lehman et al. 2020). Furthermore, the use of eDNA screening also enables the early detection of alien and invasive species, which is of paramount importance for their effective management and control (James et al. 2024; Thomas et al. 2020).

A total of 10 distinct rapid DNA/eDNA‐based ID tools were used across the 68 studies (see Table 1 for explanations of each tool), with multiplex PCR being the most commonly used (*N* = 23), followed by taxon‐specific qPCR (*N* = 13), taxon‐specific PCR (*N* = 9), PCR‐RFLP (*N* = 8), ddPCR (*N* = 5), LAMP (*N* = 4), multiplex qPCR (*N* = 3), repetitive sequence‐based PCR (rep‐PCR) (*N* = 2), RT High‐Resolution Melting PCR (RT‐HRM‐PCR) (*N* = 1) and FASTFISH‐ID (N = 1). Multiplex PCR and PCR–RFLP have been used for tissue‐based samples, whereas taxon‐specific qPCR has been used for both tissue DNA and eDNA (Figure 1C). LAMP, RT‐HRM‐PCR, multiplex qPCR, and rep‐PCR were also exclusively associated with tissue‐based applications. Only ddPCR was exclusively applied in eDNA studies. Despite being used in a few reviewed studies (*n* = 3), rep‐PCR and RT‐HRM‐PCR are not particularly reliable or repeatable methods (for a summarised review, see Box 2).

## 4 Applications and Technical Challenges in Chondrichthyan Identification

Based on the preceding quantitative assessment, this topic provides an overview of rapid DNA/eDNA‐based identification methods applied to Chondrichthyes. A summarised overview of all techniques is presented in Box 2. For clarity, the following sections focus on the most commonly used methods, as well as methodologies with promising future applications. These techniques have been adopted for species identification, monitoring and conservation due to their reliability, cost‐effectiveness and adaptability to diverse sample types (tissue DNA and eDNA).

### 4.1 Taxon‐Specific PCR

The taxon‐specific PCR technique applies a single primer set per reaction, specifically tailored to detect one target taxon. With this, taxon identification occurs through the presence or absence of the amplified fragment, usually visualised through electrophoresis or capillary systems (e.g., QIAxcel). Non‐specific amplification occurs when primers unintentionally bind to and amplify DNA from non‐target congeners or sympatric species, potentially leading to false positives. Given that, positive results are confirmed by sequencing in most studies (8 of 9 in our review). One exception used extensively validated primers to test 596 shark fins in Hong Kong (Clarke et al. 2006). Despite its utility, this method can yield false positives or lack taxonomic resolution, as seen with dusky shark primers unable to distinguish congeneric species (Pank et al. 2001; Clarke et al. 2006). To improve efficiency and resolve mixed samples, the use of multiple taxon‐specific markers in a single reaction has become increasingly common in trade monitoring.

### 4.2 Multiplex PCR

Multiplex PCR has emerged as the most streamlined and cost‐effective genetic identification method for identifying taxa from tissue samples. This technique uses multiple pairs of taxon‐specific primers in a single reaction tube to produce taxon‐specific fragment sizes, requiring no downstream processing (da Silva Ferrette et al. 2019). Furthermore, it has proven highly applicable to processed products (e.g., dried fins; Abercrombie et al. 2005). Despite these advantages, important factors such as annealing temperature, primer and reagent concentrations and enzyme fidelity must be carefully adjusted to prevent non‐specific amplifications (Henegariu et al. 1997; Hulley et al. 2019). The addition of both positive and negative controls is essential to ensure the reaction works properly, since positive controls confirm that the assay can successfully detect the target DNA, while negative controls help detect contamination or false positives (Bustin 2004). Primer specificity testing, especially on a range of potentially sympatric taxa, and sequencing a subset of positive multiplex PCR products are recommended to ensure reliable assay design and validation (Caballero et al. 2012).

### 4.3 PCR–RFLP

When viable, techniques that do not rely on multi‐primer reactions could be a good alternative to primer development and testing multiple sets of primers, which can be time‐ and cost‐consuming (Mendonca et al. 2009; Rasmussen and Morrissey 2008). One such alternative is PCR–RFLP, which relies on restriction enzymes to produce taxon‐specific fragment patterns. First, a DNA fragment is amplified through PCR using universal primers. Second, one or more restriction enzymes are added to digest the amplified fragment at a specific recognition site, generating a distinct digestion profile (i.e., each taxon will have one or more DNA fragments with distinct sizes) (Johny et al. 2022; Rasmussen and Morrissey 2008). For instance, the enzyme *MboI* produced five distinct profiles from a 750 bp fragment of the COI gene based on at least five restriction sites to differentiate swordfish from five shark species (Ferrito et al. 2019). The discriminatory resolution of PCR–RFLP depends on the location of restriction site polymorphisms among different species, which means that a single informative polymorphism within a small region can create or eliminate a recognition site (Kratochwil et al. 2022; Rasmussen 2012), making PCR–RFLP particularly suitable when genetic divergence is minimal (e.g., closely related taxa, intraspecies variability, highly conserved regions) (Canfield and Bowen 2021; Dey et al. 2025; Rasmussen 2012). The architecture of PCR–RFLP facilitates the development of assays, as it eases the need for multiple primers but limits the targets identified to the availability of recognition sites that depend on species‐specific mutations (Rasmussen 2012; Rasmussen and Morrissey 2008). On the other hand, it requires a thorough *in silico* assessment and empirical validation of restriction enzymes across multiple potentially sympatric taxa (Sabir et al. 2019; Kratochwil et al. 2022).

### 4.4 Taxon‐Specific qPCR

In contrast to conventional PCR approaches, which only indicate species presence or absence, quantitative methods such as qPCR offer increased sensitivity and the ability to estimate the quantity of target DNA. This is achieved by measuring fluorescence during amplification and comparing it to a standard curve derived from known reference concentrations (Pont et al. 2023). Taxon‐specific qPCR has been frequently applied in Chondrichthyes research, especially for detecting species from eDNA samples (Figure 1C). While taxon‐specific qPCR can be performed with intercalating binding dyes (e.g., SYBR Green), probe‐based assays with fluorophore‐labelled oligonucleotides (e.g., TaqMan probes) are often preferred in eDNA applications, particularly when distinguishing closely related taxa (Klymus et al. 2020; Wilcox et al. 2013). Unlike primers, which are essential for amplification, probes are optional but improve specificity and sensitivity by requiring an additional sequence match within the target DNA (Gargan et al. 2017; Herder et al. 2013). This is particularly important when mismatches in primer binding sites alone fail to prevent non‐target amplification, as demonstrated in the taxon‐specific eDNA assay designed for detecting the Sicklefin Devil Ray, *Mobula tarapacana* , where the addition of a probe was necessary to achieve species‐level specificity (Gargan et al. 2017). Nonetheless, probe‐based qPCR can be costly, as it requires additional reagents to accommodate technical replicates and standard curves (typically generated from synthetic DNA such as gBlocks) to ensure reliable data interpretation as part of robust validation protocols (Pont et al. 2023).

### 4.5 Multiplex qPCR

To simultaneously test for multiple species, multiplex qPCR represents a suitable alternative to traditional single‐target assays, combining the theoretical basis of multiplex PCR with the advantages of a quantitative approach, as previously discussed in qPCR. Among the assays available for Chondrichthyes identification, the multiplex qPCR assay developed by Cardeñosa et al. (2018) has gained notable prominence due to its efficiency. This assay identified nine CITES‐listed shark species and enabled the processing of 94 samples within four hours, demonstrating strong potential for large‐scale identification of tissue samples. It was also applied in three on‐site tests in Hong Kong to demonstrate the portability of the approach, where a portable qPCR thermocycler was used to screen shark fins, leading to the detection of CITES‐listed species. Portable qPCR devices such as the QuantStudio 5 System (Thermo Fisher) and the Franklin Real‐Time Thermocycler (Biomeme) have shown promise as on‐site monitoring tools for both tissue‐based and eDNA applications (Trujillo‐González et al. 2022; Prasetyo et al. 2023). Interestingly, multiplex qPCR was applied to one eDNA study that developed an assay to simultaneously detect three shark species commonly associated with human–shark conflicts in Australia, providing a relatively rapid, cost‐effective and non‐invasive alternative to traditional survey methods for monitoring shark activity in high‐risk areas (van Rooyen et al. 2021).

### 4.6 ddPCR

Droplet digital PCR (ddPCR) is a highly sensitive method for quantifying low levels of DNA, offering greater precision than qPCR for eDNA applications. In ddPCR, the PCR reaction is partitioned into thousands of oil‐encapsulated nanodroplets, each acting as an independent reaction (Doi et al. 2015; Cao et al. 2016; Guri et al. 2024). After amplification, droplets are screened for fluorescence to determine which contain the target DNA. The proportion of positive droplets is then used to calculate the absolute quantity of DNA, removing the need for standard curves and reducing variability between runs (Cao et al. 2016; Wood et al. 2019). This droplet‐based partitioning, combined with endpoint detection, also helps reduce PCR inhibition, which is common in eDNA samples (Doi et al. 2015). ddPCR is particularly well suited for detecting highly threatened species present at low abundance, such as the critically endangered smalltooth sawfish ( *Pristis pectinata* ), for which the assay successfully detected approximately 0.25 pg of target DNA (Lehman et al. 2020, 2022). Although ddPCR offers greater precision and sensitivity, it is generally more time‐consuming than qPCR and requires more precise determination of fluorescence thresholds using probe‐based assays and more expensive facilities (Doi et al. 2015). These constraints likely contribute to its more limited use in Chondrichthyes eDNA applications compared to taxon‐specific qPCR (see Doi et al. 2015 for a comprehensive technical comparison with qPCR).

High assay specificity and sensitivity are essential when working with eDNA due to the low concentration and degraded quality of target DNA, as well as the presence of non‐target DNA (Klymus et al. 2020). This is especially important when a rare target species is sympatric with a more abundant and closely related taxon, which can lead to competition during amplification (Freeland 2017; Klymus et al. 2020; Tsuji et al. 2020). Nonetheless, the integration of occupancy models, especially in quantitative eDNA monitoring techniques, has proven to be a powerful framework for estimating species presence across time and space while explicitly accounting for imperfect detection (Dorazio and Erickson 2018; Wood et al. 2019). These models use detection/non‐detection data collected from replicates to estimate the probability that a species is truly present, even when not detected in every sample (Dorazio and Erickson 2018).

### 4.7 LAMP

One of the fastest DNA tools for Chondrichthyes identification is the on‐site technique LAMP (But et al. 2020). LAMP amplifies DNA at a constant temperature using a DNA polymerase with strand displacement activity, which allows strand separation and synthesis to occur simultaneously without thermal cycling (Nagamine et al. 2002; Notomi et al. 2000). The method uses at least two pairs of primers that bind to four distinct regions of the target DNA, forming a loop structure that accelerates amplification and enhances specificity (Nagamine et al. 2002). Unlike traditional PCR‐dependent techniques, LAMP does not rely on a thermocycler with fluorescence‐based, or UV‐based detection, making it particularly well suited for field‐based species identification and law enforcement applications (Lin et al. 2021; Tiktak et al. 2024). Assays for 12 CITES‐listed shark species found in the Hong Kong shark fin market have been successfully developed, with results obtained within 2 to 3 h for 14 samples (But et al. 2020). Moreover, a refined multiplex LAMP assay targeting three CITES‐listed shark species was developed, which reduced the diagnostic period to 1 h but increased costs (Lin et al. 2021). Although LAMP provides visual readouts without specialised equipment, its accuracy depends on the careful design of multiple primers to prevent non‐specific amplifications as well as specific reagents, which makes the design and optimisation of the technique more time‐consuming (Moehling et al. 2021; Notomi et al. 2015; Tomita et al. 2008).

LAMP has also been used to detect species in eDNA samples (Nakajima et al. 2024; Porco et al. 2022; Williams et al. 2017), but its application to Chondrichthyes eDNA remains unexplored. The effectiveness of LAMP in environmental samples is hindered by challenges such as low DNA abundance and the presence of inhibitors—such as humic and tannic acids, sediment particles, metals and dissolved ions. These issues also affect other eDNA‐based ID tools such as taxon‐specific qPCR that is similarly susceptible to inhibition (Mauvisseau et al. 2019). Furthermore, the specificity of LAMP in eDNA applications may be compromised in the presence of closely related species, requiring in‐depth validation to avoid false positives (Kim et al. 2023).

## 5 Current Resources for Chondrichthyan Molecular Identification and Monitoring

### 5.1 Applied Molecular Markers

**FIGURE 2:**
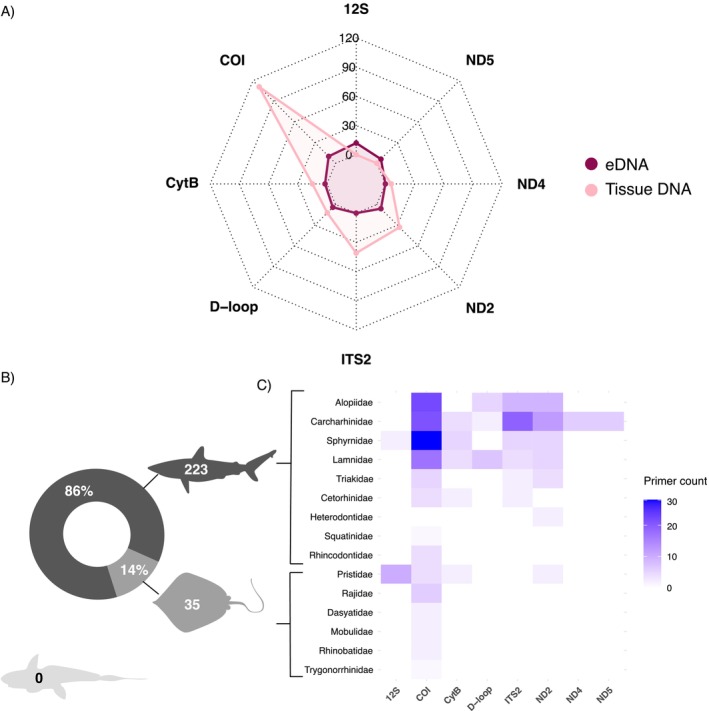
Overview of taxon‐specific primers designed for Chondrichthyes detection. (A) Total number of primers developed across the eight molecular markers identified for tissue DNA and eDNA applications. (B) Proportion of taxon‐specific primers between sharks, rays and chimaeras. (C) Heatmap organised by family illustrating the abundance of taxon‐specific primers across the eight molecular markers.

The 258 primers collectively reported in the 68 focal studies were designed to amplify taxon‐specific regions from eight loci—seven mitochondrial (COI, Cytb, D‐loop, 12 s rRNA, NADH2, NADH4 and NADH5) and one nuclear (ITS2) (Figure 2A)—primarily targeting shark species (Figure 2B). Mitochondrial DNA (mtDNA) has been applied extensively in species‐level identification analyses because it is haploid and abundant in cells, which is particularly important in eDNA assays, given the typically low quantity of target DNA in the environment (Klymus et al. 2020). Additionally, certain mitochondrial regions strike a useful balance for genetic identification, being sufficiently conserved within species to enable taxon‐specific primer design, yet variable enough between species to minimise non‐specific amplification in non‐target taxa (Guo et al. 2022). Nonetheless, the general preference for mitochondrial markers, especially COI, may be driven by the greater availability of mtDNA reference sequences in Chondrichthyes (Alvarenga, Bunholi, et al. 2024; Domingues et al. 2021), a pattern which has also been observed across other rare taxa (Duarte et al. 2023).

Tissue‐based identification was most commonly achieved with Cytochrome Oxidase I (COI) primers (*N* = 121 primers), followed by the NADH dehydrogenase subunit 2 (NADH2) primers (*N* = 39 primers) (Figure 2A; Appendix S2, Table S8). Both regions are among the most widely used in rapid DNA/eDNA‐based ID tools and have been applied across multiple shark and ray families (Figure 2C). While COI is a popular marker for fish and sharks due to its high diversity across species (Hebert et al. 2003; Ward et al. 2005), NADH2 has also been used to detect Chondrichthyes trade (Canfield and Bowen 2021; Farrell et al. 2009). In Chondrichthyes, the faster rate of evolution in NADH2 compared to COI helps address taxonomic uncertainties, especially for morphologically similar species (Naylor et al. 2012). For example, the relatively large number of NADH2 nucleotide differences among congeneric species has allowed the design of taxon‐specific primers for rapid techniques, such as LAMP, when targeting species that co‐occur with congenerics, such as the Silky Shark ( *C. falciformis* ) and the Oceanic Whitetip Shark ( *Carcharhinus longimanus* ) (But et al. 2020). The nuclear multi‐copy region ITS2 has also been widely used in identification studies (*N* = 41), and it can differentiate at least some shark species (Abercrombie et al. 2005; Domingues et al. 2013; Shivji et al. 2002; Nachtigall et al. 2017; Tiktak et al. 2024).

In eDNA applications, 12S rRNA (*N* = 12) and COI (*N* = 10) regions are the most widely used (Figure 2A; Appendix S2, Table S8), similar to patterns observed across other aquatic vertebrates (Duarte et al. 2023). The 12S rRNA region is generally well conserved across shark species, allowing for broad‐range primer design (But et al. 2020), yet it also exhibits sufficient sequence divergence among closely related species to enable taxon‐specific assays. For example, multiple 12S rRNA assays have been developed to distinguish sawfish species, with primer and probe sets incorporating five to 11 cumulative mismatches relative to the nearest non‐target sawfish species, providing the necessary specificity for reliable identification (Cooper et al. 2021). In contrast, for closely related devil rays, COI was the only region that allowed the design of primers producing a sufficiently short and variable amplicon to enable the exclusive detection of *M. tarapacana* eDNA (Gargan et al. 2017)—an important consideration when working with degraded environmental samples. Mitochondrial DNA markers dominate current taxon‐specific eDNA applications; nuclear markers are not commonly used due to their lower cellular abundance, which is further diluted in environmental samples (Duarte et al. 2023). To date, they have not yet been used in eDNA‐based rapid tools for Chondrichthyes.

### 5.2 The Importance of Curated Reference Databases

For any taxon‐specific DNA or eDNA‐based identification tools, the success of molecular markers relies heavily on assay sensitivity (the ability to detect low quantities of target DNA) and specificity (the ability to amplify only the DNA of the target species). Both depend on the careful design and *in silico* testing of primers against sequences from the target species as well as closely related or co‐occurring species to ensure accurate detection (Budd et al. 2021; Klymus et al. 2020; Appleyard et al. 2025). This requires a comprehensive and well‐curated sequence database that also reflects intraspecific variation, which might be challenging for markers other than COI, which do not have a dedicated global source such as the Barcode of Life Data Systems (BOLD). Nevertheless, the growing use of 12S rRNA in eDNA metabarcoding applications targeting entire fish communities, including elasmobranchs, has helped fill gaps in 12S rRNA in sequence databases. As researchers build increasingly complete sequence databases to improve taxonomy assignment (Bunholi et al. 2023; Miya 2022), these efforts will also indirectly benefit the design of taxon‐specific assays. Notable initiatives to improve current databases include the GAPeDNA platform, which compiles and maps global species records across five molecular markers (12S, 16S, 18S, COI and CytB) (Marques et al. 2021). A comprehensive evaluation of available Chondrichthyes sequences in public databases (including associated geographic metadata) would support the selection of appropriate markers for effective taxon‐specific assay design and inform decisions on where and which species require additional mtDNA sequencing efforts (Lim and Thompson 2021).

### 5.3 How Well Are Chondrichthyans Represented in the Available Resources?

Most of the primers we identified were developed for elasmobranchs (shark *N* = 223 = 86.4%, and ray *N* = 35 = 13.6%) with no primers specifically targeting holocephalans (i.e., chimaeras and ratfish) (Figure 2B). Despite being overlooked in DNA‐based research, chimaeras are increasingly captured in some coastal and many deepwater fisheries as both target and bycatch (Finucci et al. 2021), which raises concerns about potential risks of local overfishing and their overall conservation status due to a lack of knowledge about this group (Finucci et al. 2021). Overall, we identified only three studies on holocephalans that employed genetic tools: two investigated the population genetic structure of the rabbitfish *Chimaera monstrosa* (Carugati et al. 2024; Catarino et al. 2017) and the other investigated the population structure and demographic history of the American elephantfish *Callorhinchus callorynchus* (Erk et al. 2025). All three studies relied on COI sequencing and did not apply rapid DNA/eDNA‐based identification tools. Within elasmobranchs, rays have received less attention than sharks in genetic research. While often considered of less economic value, some batoid groups like the guitarfishes have high commercial value but remain overlooked in public interest and research funding compared to sharks (Chatzispyrou and Koutsikopoulos 2023; Ebert 2017). Furthermore, many ray species are caught as unreported bycatch, posing a significant threat to this group and emphasising the importance of taxon‐specific monitoring tools (Cashion et al. 2019; Ferrette et al. 2019).

The taxon‐specific primers developed for tissue DNA and eDNA applications currently comprise 57 species, 23 genera, 15 families and nine orders (Appendix S2, Table S9), representing 4.5% of the assessed Chondrichthyes by IUCN (IUCN 2025) and 35.4% of the Chondrichthyes species listed in CITES Appendices (CITES 2025). The top five families with taxon‐specific primers were Carcharhinidae (*N* = 58, 27.6%), Alopiidae (*N* = 45, 17.7%), Sphyrnidae (*N* = 45, 17.7%), Lamnidae (*N* = 37, 14.6%) and Pristidae (*N* = 18, 7.1%), with the latter being the only family of rays included in the top five (Figure 2C). Scalloped Hammerhead (*Sphyrna lewini, N* = 24, 10%) and Bigeye Thresher ( *Alopias superciliosus* , *N* = 19, 7.9%) sharks had the highest numbers of taxon‐specific primers developed (Appendix S2, Table S9), which could be due to their circumglobal distribution and higher prevalence in fin trade surveys (Cardeñosa et al. 2023; Clarke et al. 2006). Out of the 46 shark species for which taxon‐specific primers have been developed, 38 (82.6%) are listed as threatened on the IUCN Red List of Threatened Species, and 39 (84.7%) are listed in CITES (see Appendix S2, Table S9). Additionally, 29 (63%) of these species have been identified in Hong Kong shark fin markets (Cardeñosa et al. 2023). All 11 ray species with taxon‐specific primers are assessed as either critically endangered or endangered in the IUCN Red List, and six (54.5%) are currently listed in CITES (Appendix S2, Table S9).

## 6 Recommendations for Future Investigations and Applications

To enhance the effectiveness and accessibility of genetic monitoring for sharks and rays, future research should focus on (i) expanding and refining the application of existing rapid DNA/eDNA‐based ID tools (e.g., multiplex PCR, taxon‐specific qPCR and ddPCR); (ii) generating essential support resources, such as comprehensive sequence reference databases, which are critical for taxon‐specific primer design—this is particularly urgent for rays (e.g., guitarfishes), given their high vulnerability to bycatch and endangered status. Efforts should also prioritise expanding reference databases for mtDNA regions beyond COI, as robust assay development requires testing and validation across multiple regions, and (iii) developing novel rapid assays to identify as many Chondrichthyes species as possible—prioritising understudied and threatened species—for real‐time monitoring, particularly in regions of high conservation concern or active shark trade. Enhancing the sensitivity of eDNA tools will further improve the detection of rare species, overcoming challenges like low DNA abundance and environmental inhibitors. Furthermore, cost‐effectiveness and accessibility must be addressed, especially in the Global South. Advancing low‐cost alternatives like LAMP and portable DNA analysis devices can make genetic monitoring feasible in resource‐limited settings, allowing for field‐based, real‐time species identification. Additionally, the development of comprehensive databases for taxon‐specific primers and protocols is essential to ensure the widespread and effective use of these tools. Expanding research efforts in understudied biodiversity hotspots (e.g., Southeast Asia, South Africa, the Brazilian coast and Madagascar) may also increase the global applicability of genetic monitoring methods and improve biodiversity knowledge for Chondrichthyes. Finally, integrating rapid DNA/eDNA‐based ID tools into enforcement and management strategies whenever possible can significantly contribute to long‐term conservation success. Incorporating these tools into fisheries management and national/international monitoring programmes, and expanding their applications for regulation enforcement purposes, should strengthen enforcement against illegal fisheries and trade and support better‐informed conservation decisions.

## Author Contributions

M.M.A. conceived the project idea; M.M.A., I.V.B., P.C., V.P.d.C. and A.M.S.‐C. conceptualised the manuscript; M.M.A. and I.V.B. developed the methodology and administered and supervised the project, with guidance from P.C., V.P.d.C., R.R.D. and A.M.S.‐C.; I.V.B. and M.M.A. performed formal analysis; M.M.A., I.V.B., P.C., V.P.d.C. and R.R.D. performed investigation and visualisation; M.M.A., I.V.B., P.C., V.P.d.C., A.M.C.L.A., C.P., M.M.C., E.V.d.J., L.M.F., Y.T., D.S. and M.E.L.L. performed data curation, with validation by M.M.A., I.V.B., A.M.C.L.A., C.P., M.M.C. and E.V.d.J.; P.C. contributed to funding acquisition; I.V.B., M.M.A., V.P.d.C., P.C., R.R.D. and E.V.d.J. wrote the manuscript; M.M.A., I.V.B., P.C., R.R.D., L.M.F., C.P., A.M.S.‐C., V.P.d.C. and Y.T. reviewed and edited the manuscript. All authors read and approved the final version.

## Conflicts of Interest

The authors declare no conflicts of interest.

## Supporting information


**Data S1:** Appendix 1.

**Data S2:** Appendix 2.

## Data Availability

All datasets and pipelines supporting this study are available on GitHub at https://github.com/Bunholi/DNA_rapid_tools. A full catalogue of rapid tools and primers available up to March 2025 is provided in Appendix S1. Additionally, a detailed description of the methodology used to obtain and analyse the data is presented in Appendix S2. To support knowledge sharing, we developed an e‐book and flyer explaining Rapid DNA/eDNA‐based ID Tools in clear language to help practitioners integrate them into their workflows. These resources, useful beyond Chondrichthyes, will be available in English, Portuguese, Spanish, and French–covering most of the Global South languages, the ones who need this support the most–with a translation template upon request. This research was conducted entirely by Brazilian scientists, from one of the Global South countries that will benefit from the resources gathered here. By providing fast, cost‐effective species identification methods, this study contributes to improved management and conservation strategies. No specific permits were required to conduct this research.
