# Supplementary material for: Rapid DNA/eDNA‐Based ID Tools for Improved Chondrichthyan Monitoring and Management
Source: Mol Ecol Resour. 2025 Sep 19;25(8):e70044. doi: 10.1111/1755-0998.70044 (PMC12550492; doi:10.1111/1755-0998.70044)
Supplement: Supplementary file 1 — Data S1: Appendix 1. [file MEN-25-e70044-s001.pdf]

MOLECULAR ECOLOGY RESOURCES

Supplemental Information for:

Linking Rapid DNA-based tools to practitioners for improved Chondrichthyes management and conservation

SUPPLEMENTARY METHODS

Data collection and treatment

A comprehensive literature review was conducted following the RepOrting Standards for Systematic Evidence Syntheses (ROSES; Haddaway et al. 2018). An extensive Boolean search (AND, OR, NOT) was performed on Web of Science to identify peer-reviewed studies that developed or applied Rapid DNA-based tools (both laboratory-based and on-site techniques) or primers for identifying Chondrichthyes species. Keywords related to DNA-based tools and to other techniques that may apply species-specific primers were searched in the topic field (keywords, title, and abstract), while those related to Chondrichthyes were searched specifically in the title field. Studies containing keywords unrelated to Chondrichthyes research were excluded (Table S6).

Table S6. Keywords applied at Web of Science for literature search.

Web of Science Search		
	Topic	Primer* OR "PCR" OR "PCR*Multiplex" OR "Multiplex*PCR" OR "PCR*RFLP" OR "Multiplex*RT*qPCR" OR "RT*qPCR*Multiplex" OR "qPCR" OR "Real Time PCR" OR "RT*qPCR" OR "Droplet Digital PCR" OR "ddPCR" OR "Nested*PCR" OR "nPCR" OR "Lab*on*a*Chip*Device*" OR "LOC" OR "LAMP" OR "LAMP*PCR" OR "PCR*LAMP" OR "NASBA" OR "Biosensor*" OR "Gene Probe Technology" OR "Gene Chip Technology" OR "eDNA" OR "eRNA" OR "eDNA*qPCR" OR "eRNA*qPCR" OR "metabarcoding" OR "barcoding" OR "high*resolution melting" OR "HRM" OR "Sanger*sequencing" OR "DNA*sequencing" OR "sequencing" OR species specific diagnostic nucleotides OR species specific primer* OR species specific probe* OR microarray* OR "DNA chip" OR specific primer* OR "MinION" OR "nanopore sequencing" OR "nanopore" OR "environmental DNA*"
AND	Title	elasmobranch* OR "chondrichth*" OR "shark*" OR "ray" OR "rays" OR "guitarfish*" OR "guitar-fish*" OR "stingray*" OR "skate*" OR "skateray*" OR "skate ray*" OR "rhinoray*" OR "sawfish*" OR "chimaer*" OR "ghost shark*"
NOT	All Fields	"x ray*" OR "x-ray*" OR "UV-ray*" OR "UV ray*" OR "radiation*" OR "irradiation*" OR "ultraviolet" OR "radiograph*" OR "Ray*fluid*" OR "ray's fluid" OR "integrative taxonomy" OR "ray-finned" OR "ray-finned fish*" OR "ray*finned*fish*" OR "blue ray*" OR "ultra violet ray*" OR "blu-ray" OR "γ-rays" OR "UV lamp*" OR "ultra-violet ray*" OR "gamma ray*" OR "gamma-ray*" OR "cosmic ray*" OR "mycobacter*"OR "chimerism*"

The final dataset comprised 742 studies, which underwent manual screening based on three inclusion criteria: (1) relevance to Chondrichthyes research, (2) development or application of specific primers for taxonomic identification, and (3) development or application of Rapid DNA-based tools for taxonomic identification. Each study was independently evaluated by three reviewers and included if at least two agreed (majority rule). The reliability of this review process was assessed using inter-rater agreement (IRR), a metric for consistency among independent reviewers (Gisev et al. 2013). The screening identified 352 studies focusing on specific primers (primer dataset), achieving an IRR of 0.766. Among these, 86 studies involved DNA-based rapid tools (rapid tools dataset), with an initial IRR of 0.554. To improve reliability, two reviewers reassessed the rapid tools dataset under a full agreement criterion, confirming 58 studies and improving the IRR to 0.927. One study was inaccessible, and another, which utilized microarray tools not tested in situ (Vargheese et al. 2019), was excluded. Additionally, two studies published before 2000 were removed to ensure relevance to contemporary techniques.

The snowballing method (Wohlin 2014) was applied to expand the rapid tools dataset, incorporating both forward (citing) and backward (cited) references. This iterative process was concluded when no new studies were identified, yielding 16 additional studies. The final rapid tools dataset included 68 studies, comprising 62 laboratory-based techniques (Table S1) and six on-site identification tools (Table S2).

From the broader dataset of 367 studies, primer information was compiled, including sequences and names, to create a comprehensive database. Duplicate primers were initially identified and excluded, resulting in 423 unique primers. Further manual curation removed irrelevant primers (e.g., universal primers not critical to DNA-based tools), resulting in a final set of 303 primers. The curated primer database was categorized into specific primers (Table S5) and universal primers (Table S6). Data on primer and probe development (e.g., GC content, melting temperature) and primer specificity were included to enhance the usability of these resources. Additionally, a separate dataset of probes was documented, including their characteristics and taxonomic scope (Table S7). Geographic information was extracted from the rapid tools dataset, including sample origins and author affiliations, along with details of the techniques applied (e.g., type of tool, sample targets) (Table S2).

Data analysis

Data wrangling, filtration, and visualization were performed using the R packages *tidyverse*, *stringr*, *ggplot2*, *maps*, *packcircles*, and *cowplot* in R version 4.2.1 (R Core

Team, 2022). Metadata and R scripts are available on GitHub (https://github.com/Bunholi/DNA_rapid_tools).

SUPPLEMENTARY FIGURES

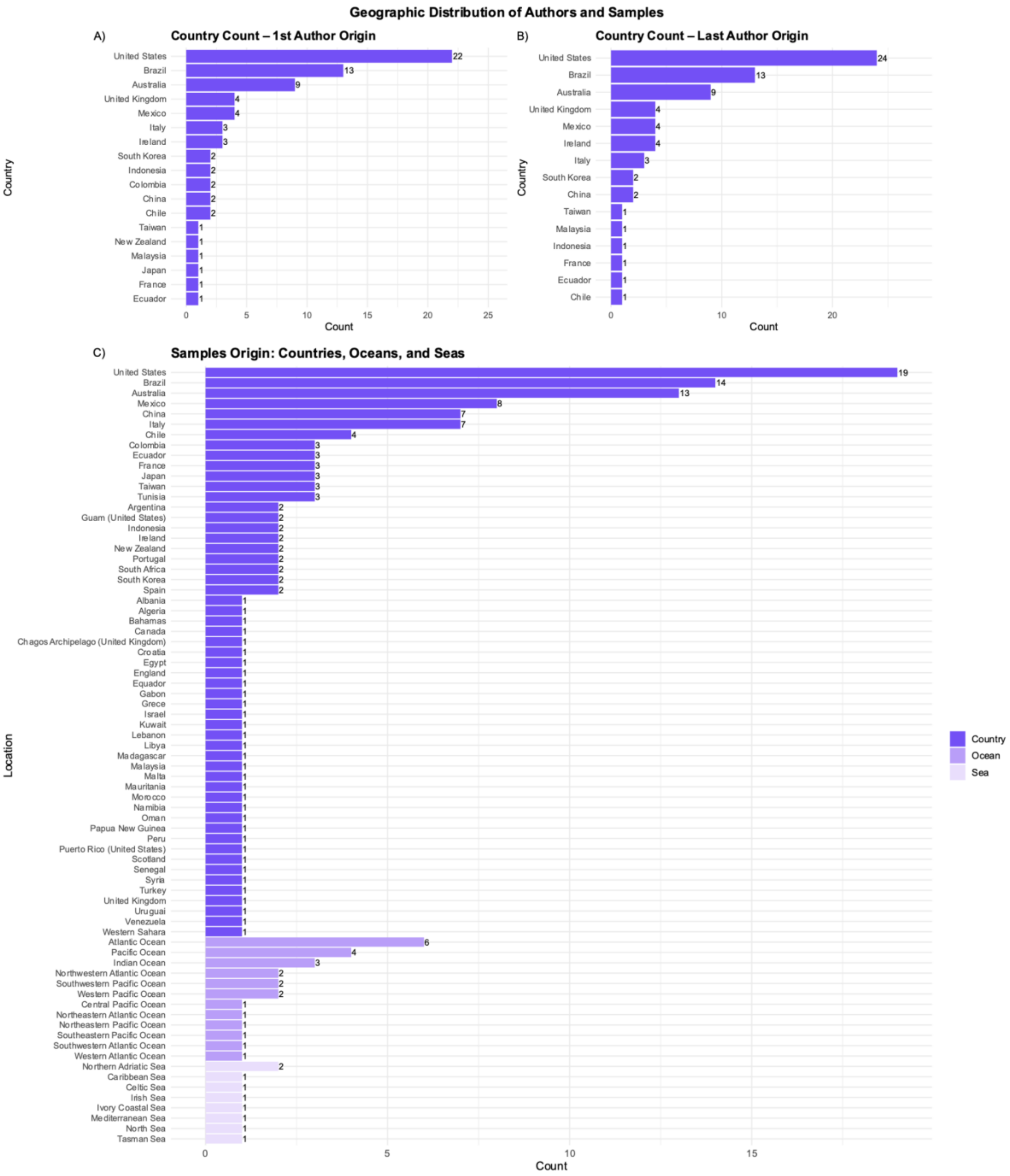

FIGURE S1. Geographic distribution of authorship and samples. Affiliation of A) first and B) last authors from the retrieved studies, together with the C) Origin of collected samples.
